# A Peptide Family Being Re-United: The Angiotensins Coming in from the Cold

**Published:** 1992-08

**Authors:** Edward C. Osborn, Andrew Davenport, Allen E. Goodship, J. Campbell Mackenzie

**Affiliations:** Department of Renal Medicine, Southmead Hospital, Westbury-on-Trym, Bristol BS10 5NB; Department of Renal Medicine, Southmead Hospital, Westbury-on-Trym, Bristol BS10 5NB; Department of Anatomy, School of Veterinary Science, University of Bristol, Bristol BS1 5LS; Department of Renal Medicine, Southmead Hospital, Westbury-on-Trym, Bristol BS10 5NB

## Abstract

The renin-angiotensin-aldosterone system is now considered to be far more complex than previously thought when the vasoconstrictor and other physiological effects of the octapeptide angiotensin II in the circulating blood were emphasized. The reasons for this altered viewpoint, involving angiotensins other than the octapeptide in the regulation of blood pressure, water and electrolyte homeostasis, are briefly advanced and discussed.


### A Peptide Family Being Re–united: the Angiotensins Coming in from the Cold

**Edward C. Osborn, Ph.D.,**
**Andrew Davenport, M.D.(Cantab).,**
**Allen E. Goodship, Ph.D.***
**J. Campbell Mackenzie, F.R.C.P.(Ed.)**
Department of Renal Medicine,
Southmead Hospital, Westbury–on–Trym, Bristol BS10 5NB
and
*Department of Anatomy,
School of Veterinary Science,
University of Bristol, Bristol BS1 5LS

### SUMMARY
The renin-angiotensin-aldosterone system is now considered to be far more complex than previously thought when the vasoconstrictor and other physiological effects of the octapeptide angiotensin II in the circulating blood were emphasized. The reasons for this altered viewpoint, involving angiotensins other than the octapeptide in the regulation of blood pressure, water and electrolyte homeostasis, are briefly advanced and discussed.

### INTRODUCTION
The initial observation that the kidney contains a highly effective pressor substance was made almost a century ago.[1] It was not until 1934, however, when Goldblatt and his colleagues demonstrated that constriction of the renal artery in dogs produced hypertension,[2] that there was an explosion of both scientific and clinical interest in the relevant renin-angiotensin (RA) system. The first cure for hypertension by nephrectomy was reported soon after,[3] an event that immediately led to widespread surgical management of renovascular hypertension, as evaluated by Homer Smith in the 1950's.[4] Present day therapeutic approaches in such hypertension have lately been extensively reviewed by Hollenberg[5] with regard to use of drugs designed to inhibit, chemically, angiotensin converting enzyme - ACE (which converts the decapeptide angiotensin I to the octapeptide angiotensin II) and also with surgical intervention and/or angioplasty which produce the best results — in terms of both effectiveness and risk — in patients with fibro-muscular dysplasia; the least favourable outcome occurred in cases of widespread advanced atherosclerosis.

There was almost universal agreement until quite recently that RA activity was, to all intents and purposes, entirely mediated by the octapeptide angiotensin II, after its removal from the bloodstream in the systemic circulation, **via** its vasopressor and other biological effects. In this earlier scenario the decapeptide angiotensin I was regarded as the inactive precursor of the octapeptide with ACE activity being essentially confined to the lungs. The estimation of the plasma concentrations of angiotensin II and of the enzyme renin (which acts on its substrate angiotensinogen — a tetradecapeptide — to produce the decapeptide)[6] thus became of overriding interest. As a result other possible modes of action of the RA system received little attention. Such a simplistic viewpoint is, however, no longer tenable so that the relevant homeostatic mechanisms involved in blood pressure control, and water and electrolyte balance, are undergoing a profound reappraisal[6-10] both regarding RA and RAA (that also involves the steroid aldosterone) activity.

### ANGIOTENSIN I: THE LONG NEGLECTED FAMILY MEMBER MAKES A COMEBACK
Until lately most investigators felt that the decapeptide was biologically inactive. However, as suggested in 1987,[10] intra-renal effects of angiotensin I could promote an antidiuresis mediated **via** the peritubular capillaries, this effect involving complex relationships between angiotensins I and II within the kidney; these importantly concern the distribution of pressures throughout the renal vasculature,[7, 8] and also the intra-renal pattern of sodium chloride levels which strongly influences the ability of the decapeptide to promote water reabsorption into the circulating blood.[11] Decreased (NaCl) also stimulates antidiuretic hormone (ADH, vasopressin) secretion by the pituitary[7, 8] and this, coupled with the intra-renal effects of angiotensin I,[7, 8, 11] can profoundly affect renal venous plasma (solute) together with the relative plasma content of blood in the renal vein.

The increased awareness of the possible effects of angiotensin I in blood pressure regulation, combined with the improved availability and acceptability of radioactive iodinated angiotensin derivatives, and the development of more specific radioimmunoassay techniques, will doubtless be the basis of investigations supplementing the kind of clinical study involving the decapeptide that has recently been reported.[12]

### THE APPARENT CLAIMS OF ANGIOTENSIN III
The loss of asparagine and aspartic acid respectively from the amino-terminals of the widely available synthetic asparaginyl[1]-valyl[5]-angiotensin II (**Hypertensin**, Ciba-Geigy) and that of the octapeptide occurring in man (aspartyl[1]-**iso**leucyl[5]-angiotensin II) gives rise to heptapeptide derivatives (ie angiotensin)[2-8] designated angiotensin III. Despite the heptapeptides having only about one-third the pressor potency of the octapeptides, both the octa- and the hepta- peptides are equally effective (although the effect of angiotensin II on adrenal steroidogenesis is independent of the heptapeptide as an intermediate)[13] in stimulating aldosterone release from the adrenal zona glomerulosa.[13] (The question of distinct receptors for angiotensins II and III in the zona glomerulosa is of more than academic interest. Goodfriend and Peach,[14] suggested the possibility of receptors other than those for angiotensin II and this may provide a possible explanation for Bartter's syndrome in which the loss of sensitivity to the pressor effects of angiotensin is not paralleled by a comparable failure of aldosterone secretion. This condition is associated with persistently high circulating plasma renin levels in spite of the lack of aldosterone response and may be due to a defect that is restricted to receptors for the octapeptide).

Infusion of angiotensin II into conscious rabbits,[15] anaesthetised[16] and conscious[17] sheep causes an immediate increase in the plasma concentration of potassium ions with a simultaneous decrease in that of $Na+$ and $Cl^-$ , a pattern of (electrolyte) response that is reasonably well maintained throughout the 6 minute infusions in the sheep, with a rapid return to preinfusion plasma levels after the infusions ceased.[16, 17] The octapeptide also increases plasma ($K+$)in humans.[15] Comparable findings to those with use of the octapeptide were also obtained when the decapeptide was similarly utilized, either in sheep under general anaesthesia[16] or in the conscious animals.[7] The results clearly demonstrated that the ability of angiotensins I and II to liberate the potassium ions into the bloodstream is dose dependent[16] and unrelated to pressor sensitivity, since the conscious sheep are much more sensitive to the pressor effects of both peptides[7, 18] than are the anaesthetised ones.[16] These findings highlight the possibility that the electrolyte concentration changes are responsible, either partly or wholly, for stimulating adrenal aldosterone secretion (this being associated with increased RA activity) as initially advanced by Healy and his co-workers[15] and supported by others.[19]In both rat and dog adrenal cells the lack of any synergistic effect concerning angiotensin II and potassium suggests "that these factors share a common mode of action on steroidogenesis in zona glomerulosa cells".[13] Their dual role has been further emphasized by Laragh[20] with regard to the direct stimulation of aldosterone secretion. However Foster, Lobo and Marusic,[21] utilizing adrenal tissue from anaesthetised dogs, concluded that, because angiotension II augmented aldosterone production without concurrent alteration of either intracellular [K+] or Na,K-ATPase activity, the octapeptide does not stimulate secretion of the steroid **via** potassium release. It was, however, found that angiotensin II did not promote aldosterone synthesis below a threshold K+ concentration in the zona glomerulosa cells, although the basal aldosterone production was, itself, unaffected by low intracellular potassium concentrations **per se**.

Whether infusions of angiotensins II and III produce similar patterns of plasma (electrolyte) changes in experimental animals has apparently not yet been considered. The relevant investigations would especially concern the potassium-releasing potency of the two peptides and would doubtless throw further light on relationships between them.

### ANOTHER HEPTAPEPTIDE ON THE SCENE

The loss of phenylalanine from the carboxy-terminal of angiotensin II gives rise to the non-pressor heptapeptide **des** - phenylalanyl-angiotensin II (ie angiotensin)[1-7] and only recently has this molecule been shown to possess biological activity.[22-25] Its ability to stimulate vasopressin release equals that of the octapeptide[22] and it also modulates the sensitivity of baroreceptors as readily as does angiotensin II, as indicated.[24] This heptapeptide can be formed, either by carboxypeptidase action on angiotensin II,[24] or by endopeptidase action on angiotensin I,[23, 25] a metabolic pathway bypassing angiotensin II formation. ACE inhibition markedly increases the plasma concentration of angiotensin — (1-7) together with that of angiotensin I.[25] This importantly concerns the possibility that the heptapeptide has depressor effects in several possible ways that are augmented by ACE inhibitors as discussed by Goodfriend[24] who states that such a depressor angiotensin would be consistent with the existence of pressor, depressor and pressure-neutral members in other families of hormones.

### WHAT THE FUTURE HOLDS

The recently changed scenario regarding RA operation - coupled with doubts concerning the nature of its link-up with aldosterone secretion as outlined in this paper — will undoubtedly stimulate future developments in the relevant fields. These could well involve angiotensinogen in ways other than as a substrate for renin as indicated by Goodfriend[24]. ("For that matter, it is probably presumptious to dismiss angiotensinogen itself, and the large fragment of angiotensinogen that remains after removal of Ang I, as having no function beyond delivering its namesake.... Maybe, just maybe, angiotensinogen plus or minus Ang I is a potent protein whose other function presently eludes us"). Other possibilities regarding the tetradecapeptide could also result from the experimental findings of Poulsen and Jacobsen[26] who argued that it was a protease inhibitor restraining renin, resembling serine protease inhibitors such as $\alpha_1$-antitrypsin.

Certainly no one would now dispute either Goodfriend's statement[24] that ".... it is by no means certain that all the biologically active peptides involved in RAA operation have yet been identified" or the conclusion of Samani[9] that "We are there entering a most exciting stage in our understanding of the renin-angiotensin system and in our ability to manipulate it".[9]

### CONCLUSIONS

The rapid retreat from previous concepts of RA, RAA operation, that has resulted from investigations focussing attention on the cellular activity of the systems, has profound physiological and clinical implications. These especially concern the complex inter-relationships of the various peptides involved, in symbiotic effects influencing blood pressure control and the regulation of water and electrolyte homeostasis.

### ACKNOWLEDGEMENTS

Financial assistance by the Bristol Kidney Fund, the Sir Halley Stewart Trust, Westbury Charitable Scientific Enterprise Limited and the Dawn James Charitable Foundation is gratefully acknowledged.

### REFERENCES

1. Tigerstedt R., Bergman P. G. (1898). Niere und kreislauf. *Skand. Arch. Physiol.* **8**, 223-246.
2. Goldblatt H., Lynch J., Hanzal R. F., Summerville W. W. (1934). Studies on experimental hypertension. *J. Exp. Med.* **59**, 347-380.
3. Leadbetter W. F., Burkland C. E. (1938). Hypertension in unilateral renal disease. *J. Urol.* **39**, 611-626.
4. Smith, H.W. (1956). Unilateral nephrectomy in hypertensive disease. *J. Urol.* **76**, 685-701.
5. Hollenberg N. K. (1987). The treatment of renovascular hypertension: surgery, angioplasty, and medical therapy with converting - enzyme inhibitors. *Am. J. Kidney Dis.* **X** (Suppl 1), 52-60.
6. Osborn E. C., Mackenzie J. C., Fearn L. M. (1991). Twenty years of investigating angiotensin activity: an overview. *West Eng. Med. J.* **106**, 5-6, 8.
7. Osborn, E. C., Fearn L. M., Francis R. J., Mackenzie J. C., Mason O. F., Rigby G. V., Wilson J. (1989). Arterial plasma creatinine and K+ levels with use of exogenous creatinine and intravenously infused angiotensin I in conscious sheep. *Med. Sci. Res.* **17**, 677-679.
8. Osborn E. C., Fearn L. M., Francis R. J., Mackenzie J. C., Wilson J. (1991). Receptors for angiotensins I and II: their relevance to renal haemodynamics, blood pressure control and hind-limb blood flow. *Med. Hypoth.* **36**, 6-16 (a longer version of this paper is available on request).
9. Samani N. J. (1991). New developments in renin and hypertension. *Tissue generation of angiotensin I and II changes the picture. Br. Med. J.* **302**, 981-982.
10. Osborn E. C., Fearn L. M., Mackenzie J. C., Mason O. F., Rigby G. V., Rodgers M. W., Wilson J. (1987). Do angiotensin-induced changes in sheep renal venous blood and plasma composition indicate a reversal of glomerular filtration? *Med. Hypoth.* **23**, 137-148.
11. Osborn E. C., Mackenzie J. C., Rigby G. V., Chapman I. D. (1982). Changes in renal venous blood and plasma composition produced by injected angiotensin I in an anaesthetised sheep with hyponatraemia. *IRCS Med. Sci.* **10**, 119-120.
12. Admiraal P. J. J., Derkx F. H. M., Danser A. H. J., Pieterman H., Schalekamp M. A. D. H. (1990). Metabolism and production of angiotensin I in different vascular beds in subjects with hypertension. *Hypertension,* **15**, 44-55.
13. Catt K. J., Aguilera G., Capponi A., Fujita K., Schirar A., Fakunding J. (1979). Angiotensin II receptors and aldosterone secretion. *J. Endocrinol.* **81**, 37P-48P.
14. Goodfriend T. L., Peach M. J. (1975). Angiotensin III; (des-aspartic acid[1])-angiotensin II Evidence and speculation for its role as an important agonist in the renin-angiotensin system. *Circ. Res.* **36, 37** (Suppl I): I-38 -I-47.
15. Healy J. K., Elliott A. J., Harrison L. C. (1974). Effects of angiotensin on plasma electrolyte concentrations in rabbits and in man. *Clin. Sci. Mol. Med.* **46**, 19-36.
16. Osborn E. C., Sugden P. L., Mackenzie J. C., Aitken D.M., Chapman I. D., Howes S., Mason O. F., Rigby G. V., Wilson J. (1985). Influence of angiotensin II on the concentration of arterial plasma electrolytes in anaesthetized sheep. *J. Endocrinol.* **104**, 143-148.
17. Dyson C. E., Fearn L. M., Francis R. J., Johnson, J. L., Kendall, H.J., Mackenzie J. C., Osborn E. C., Underhill D. G. (1988). Arterial plasma electrolyte concentrations with short-term intravenous infusions of angiotensin II in conscious sheep. *Med. Sci. Res.* **16**, 817-818.
18. Bellenger C. R., Chapman I. D., Goodship A. E., Mackenzie J. C., Osborn E. C., Osborn J. E., Sugden P. L. (1982). The effects of infused angiotensin II on arterial plasma creatinine levels, using exogenous creatinine, in oonscious sheep. *IRCS Med. Sci.* **10**, 161-162.

19. Fearn L. M., James H. C. F., Mackenzie J. C., Osborn E.C. (1987). The relationship between angiotensin and aldosterone activity: the implications of angiotensin II-induced potassium release. *Med. Hypoth.* **24**, 329-330.
20. Laragh J. H. (1973). Potassium, angiotensin and the dual control of aldosterone secretion. *New Eng. J. Med.* **289**, 745-747.
21. Foster R., Lobo M. V., Marusic E. T. (1979). Studies of relationship between angiotensin II and potassium ions on aldosterone release. *Am. J. Physiol.* **237**, E363-E366.
22. Schiavone M. T., Santos R. A. S., Brosnihan K. B., Khosla M. C., Ferrario C. M. (1988). Release of vasopressin from the rat hypothalamo-neurohypophysial system by angiotensin - (1-7) heptapeptide. *Proc. Natl. Acad. Sci. USA,* **85**, 4095-4098.
23. Ferrario C. M., Barnes K. L., Block C. H., Brosnihan K. B., Diz D. I., Khosla M. C., Santos R. A. S. (1990). Pathways of angiotensin formation and function in the brain. *Hypertension,* **15** (Suppl I), I-13 - I-19.
24. Goodfriend T.L.. (1991). A family that grows from within. *Hypertension* **17**, 139-140.
25. Kohara K., Brosnihan K. B., Chappell M. C., Khosla M. C., Ferrario C. M. (1991). Angiotensin - (1-7). A member of circulating angiotensin peptides. *Hypertension,* **17**, 131-138.
26. Poulsen K., Jacobsen J. (1986). Is angiotensinogen a renin inhibitor and not the substrate for renin? *J. Hypertens.* **4**, 65-69.

